# External Sulfate Attack on Cementitious Binders: Limitations and Effects of Sample Geometry on the Quantification of Expansion Stress

**DOI:** 10.3390/ma15103677

**Published:** 2022-05-20

**Authors:** Matthias Wagner, Anne Heisig, Alisa Machner, Robin Beddoe, Detlef Heinz

**Affiliations:** 1 Professorship for Mineral Construction Materials, Department of Materials Engineering, TUM School of Engineering and Design, Technical University of Munich, Franz-Langinger-Str. 10, 81245 Munich, Germany; ma.wagner@tum.de (M.W.); anne.heisig@tum.de (A.H.); 2 Professorship for Mineral Engineering, Department of Materials Engineering, TUM School of Engineering and Design, Technical University of Munich, Franz-Langinger-Str. 10, 81245 Munich, Germany; robin.beddoe@tum.de (R.B.); heinz@tum.de (D.H.)

**Keywords:** hollow cylinder method, self-constraint, crystallisation pressure, degradation mechanism, hardened cement paste

## Abstract

The hollow cylinder method was used to estimate the expansion stress that can occur in concrete due to the crystallisation pressure caused by the formation of ettringite and/or gypsum during external sulphate attack. Hardened cement paste hollow cylinders prepared with Portland cement were mounted in stress cells and exposed to sodium sulphate solutions with two different concentrations (3.0 g L SO_4_^2−^ and 30.0 g L SO_4_^2−^). Microstructural analysis and finite element modelling was used to evaluate the experimental observations. The expansion stress calculation was verified for a range of diameter/length ratios (0.43–0.60). Thermodynamically predicted maximum expansion stresses are larger than expansion stresses observed in experiments because the latter are affected by the sample geometry, degree of restraint, pore size distribution and relaxation processes. The results indicate that differences in self-constraint at the concave inner and convex outer surfaces of the hollow cylinder lead to an asymmetric expansion stress when ettringite is formed. This leads to macroscopic longitudinal cracks and ultimately failure. Heavy structural components made of concrete are likely to support larger maximum expansion stresses than observed by the hollow cylinder method due to their self-constraint.

## 1. Introduction

The exposure of concrete to aqueous solutions, which contain sulphate ions, can lead to several chemical reactions that, among others, include the formation of ettringite and/or gypsum [1,2]. When the crystallisation of such phases occurs in confined spaces, the crystal formed can exert a crystallisation pressure on its surrounding pore walls [3], leading to expansion stress in the cement paste matrix that can exceed the tensile strength of concrete [4]. Therefore, the crystallisation pressure caused by expansive reaction products is proposed to be the main driving force of the degradation of concrete during an external sulphate attack [5,6]. The effect of the formation of gypsum on the degradation of concrete is not as obvious as the effect of ettringite, and stable gypsum might only form at high sulphate concentrations [7,8]. Therefore, the crystallisation of gypsum has not been identified as a main driving force of degradation for several cementitious systems [9,10].

The crystallisation pressure caused by the formation of ettringite or gypsum increases with the supersaturation of the pore solution with respect to ettringite or gypsum, respectively, and increases with decreasing pore diameters [3,11]. The maximum crystallisation pressure caused by ettringite formation in a particular chemical environment can be estimated by thermodynamic modelling and was determined to be about 52 MPa [6]. It is still not fully understood how the crystallisation pressure in pores translates to an expansion stress in the surrounding microstructure of the samples. Several experimental setups have been developed to measure this expansion stress [4,12,13]. One of these setups is the hollow cylinder method, which was originally developed to quantify the hygral stress in hardened cement paste [12]. In this method, a central steel rod is used to constrain the elongation of the hollow cylinder caused by the expansive process. The expansion stress σ (Pa) in the hollow cylinder can be calculated from the elongation Δl (m) of the steel rod by multiplying Δl (m) with the quotient of the stiffness constant steel rod *k* (N m^−1^) and the cross-sectional area *A* (m^2^) of the hollow cylinder (Equation (1)) [12]. The precondition for Equation (1) is that the elongation of the central steel rod is linear elastic. This can be ensured by choosing a sufficiently large steel rod diameter and consequently small degrees of expansion.

(1) $$\sigma =\left(\frac{k}{A}\right)\Delta l$$

The hollow cylinder method has been used previously to quantify the expansion stress caused by the formation of ettringite in mortar and hardened cement paste samples that were exposed to sodium sulphate solutions [4,10,14]. Earlier studies reported a maximum expansion stress of 8 MPa for mortar samples [4] and up to 13 MPa for hardened cement paste samples [14] when exposed to aqueous solutions with 30.0 g L $SO_{4}^{2-}$. For exposure to solutions with 1.5 g L SO_4_^2−^, maximum expansion stresses of 7 MPa were reported for mortar samples [4] and 8.3 MPa for hardened cement paste samples [14]. The formation of ettringite, which causes these expansive forces, occurs in pores with diameters of 10 nm to 50 nm [4]. Due to the similarity of the expansion stress reported for mortars prepared with Portland cement exposed to high sulphate concentrations and lower sulphate concentrations [4], it remains unclear whether the maximum expansion stress that has been observed for hollow cylinders could possibly be limited by the samples geometry itself.

Since the external sulphate attack is due to the ingress of sulphate from the sample surface towards the sample core, the sulphate concentration in the pore solution is larger within the outer layers of the sample cross-section, at least in the early stages of sulphate exposure. This leads to a zonal structure regarding the mineralogical composition of the sample with outer layers, in which the formation of ettringite and/or gypsum predominantly takes place [15,16,17]. As the sulphate ingress advances, the expansion stress that affects the undamaged, inner region of the sample gets closer to the core, while the size of the undamaged core itself gets smaller. It was therefore concluded that the failure of a sample by macroscopic cracking occurs if the tensile strength of the undamaged samples inner core is surpassed by the expansive stress [5].

The determination of expansion stresses caused by external sulphate attack can be a useful tool to predict the damaging potential of sulphate ingress in concrete structures. Since the relationship between crystallisation pressure, sample geometry, expansion stress and crack formation is still not fully understood, the purpose of this study was to examine the degradation process of hardened cement paste hollow cylinders [4,14] in detail. This sample geometry was chosen because it has been used in former studies to quantify the expansion stress [4,10,14] and because of the fact that hardened cement paste might lead to the determination of upper limits of expansion stress during a sulphate attack since the largest expansion pressure is likely to occur due to the formation of ettringite in small pores of the binder matrix in concrete [4]. Analysis of the microstructure as well as finite element modelling was used to address the question whether the maximum expansion stress measured by the hollow cylinder method is limited by the sample geometry itself.

## 2. Materials and Methods

### 2.1. Materials and Sample Preparation

**Figure 1:**
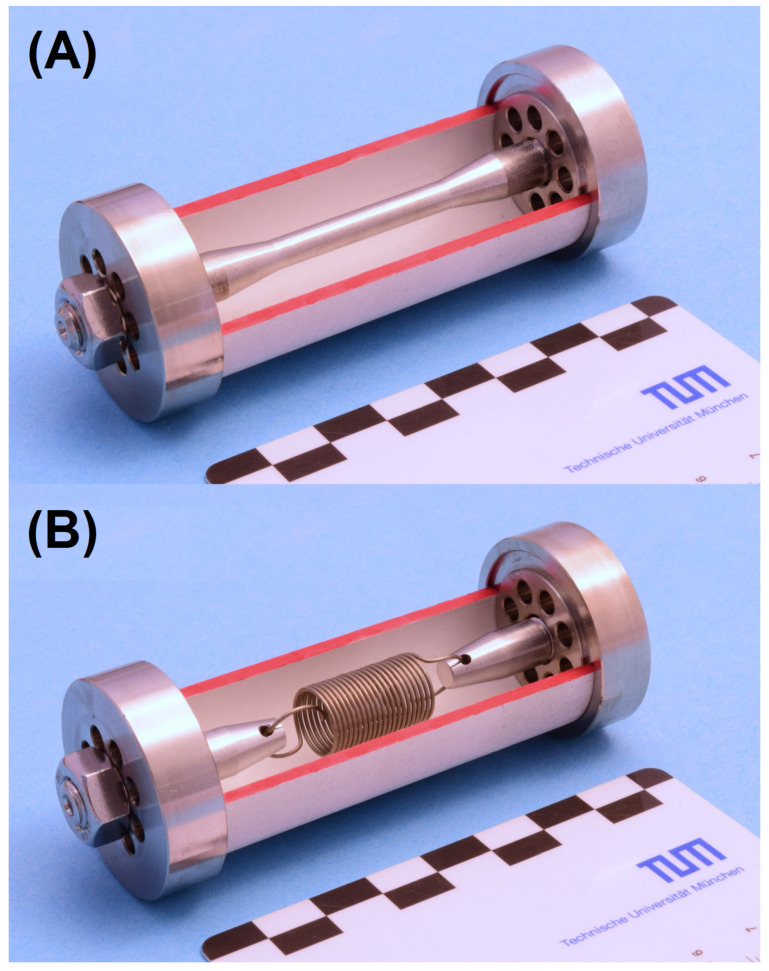
Cutaway model of the hollow cylinder stress cells used in this study. (**A**) Stress cell with constraint by a central steel rod. (**B**) Stress cell with virtually no constraint by using a steel spring with a low spring constant.

**Figure 2:**
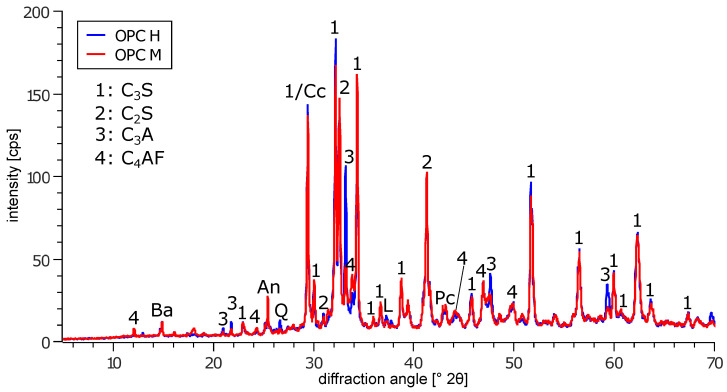
X-ray diffraction patterns of the binders used in this study. An: anhydrite; Ba: bassanite; Cc: calcite; L: lime; Pc: periclase; Q: quartz.

In this study, two Portland cements (CEM I 42.5 R) with similar CaO/$SiO_{2}$ ratios but different C_3_A contents were used to prepare hardened cement paste hollow cylinders with varying diameter/length ratios (*l* = 50 mm, 60 mm and 70 mm, Ø = 30 mm, *d* = 2.5 mm, cross-sectional area *A* = 216 mm^2^, as used in [4,10,14], Figure 1). To ensure comparability with previous hollow cylinder experiments, the range of diameter/length ratios (0.43–0.60) was chosen so that the stress cells for the original experimental setup (0.43) could be used for the entire range of hollow cylinder lengths. In order to minimise the kinetic effect of the transport of sulphate ions, the same small wall thickness (as in previous studies [4,10,14]) was used. OPC H refers to the binder with high C_3_A content (12 wt.%), and OPC M refers to the binder with lower C_3_A content (6.5 wt.%). The chemical and mineralogical composition of the cements is shown in Table 1, and the XRD patterns are shown in Figure 2. For the preparation of the cylinder paste samples, deionised water was continuously added to the dry cement under gentle stirring for 30 s. Once the complete water was added, the fresh cement paste was stirred vigorously for 60 s under partial vacuum to reduce air inclusions that could weaken the structure of the thin-walled hollow cylinders (batch size: 1.35 kg). The water/binder ratio was set to 0.5. The paste was poured into polyvinyl chloride moulds that were previously coated with a polytetrafluoroethylene spray to reduce the adhesion between the cement paste and the mould. The filled moulds were then placed in an overhead shaker at 7 rpm for 24 h after casting to reduce sedimentation during setting. For the binders used in this study, no significant shrinkage was observed that would lead to the formation of cracks within the hollow cylinders while being stored in their moulds. However, it has to be noted that a minor degree of shrinkage could possibly increase the self-constraint of the hardened cement paste to a certain degree, thus possibly leading to slightly smaller measured expansion stresses in the experiments.

The hollow cylinders were removed from their moulds after 24 h and then stored in a saturated calcium hydroxide solution until an age of 28 days and subsequently placed in stress cells, in which the expansion during storage was constrained by a central steel rod (1.4404 steel, Ø = 5 mm, *k* = 1.5 kN mm^−1^, Figure 1A) or the expansion was effectively unrestricted by using steel springs instead (1.4310 steel, *k* = 0.07 N mm^−1^, Figure 1B). The constraint exerted by the steel rod is a realistic setting for larger construction components and enables the quantification of the expansion stress (Equation (1)). The free expansion of samples in stress cells with springs represents the expansion of small laboratory samples (e.g., prisms) that possess only a minor degree of self-constraint due to their small dimensions compared to larger, real concrete structures. The end plates of the stress cells included eight through-holes each (Ø = 4 mm) to allow the advection of the fresh sulphate solution into the inner stress cell during the expansion experiments.

The initial tension of the constrained samples (central steel rod, Figure 1) was set to 3.0 MPa by applying a torque of 2.0 Nm to the outer clamping nuts.

During the preparation and measurements of the samples, it was ensured that the samples were always wet. For the sulphate exposure in the laboratory, the stress cells were fully submerged in sodium sulphate solutions with two different sulphate concentrations (30.0 g L $SO_{4}^{2-}$ (used in some laboratory sulphate resistance testing procedures, e.g., EAD 150009-00-0301 [18]) and 3.0 g L SO_4_^2−^ (lower limit of exposure class XA3 according to EN 206 [19])). The samples were stored in the solutions at 22 ${}^{{}^{\circ}}C$ until mechanical failure occurred by macroscopic cracking. At least three constrained and three non-constrained stress cells were prepared for each binder. The length changes in the central steel rods and non-constrained samples were measured every 7 days. The storage solutions were renewed after each length measurement.

### 2.2. Analytics

The chemical composition of the cements and the sodium sulphate was determined using inductively coupled plasma optical emission spectroscopy (ICP-OES, Horiba Jobin Yvon Ultima 2) after microwave digestion in nitric acid, hydrogen peroxide and hydrofluoric acid (results are reported in Table 1).

**Figure 3:**
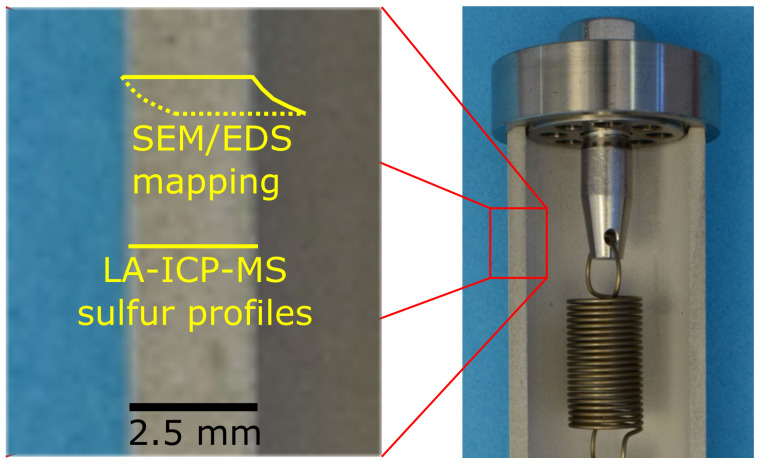
Schematic representation of the measurement ranges for SEM/EDS mapping and LA-ICP-MS sulphur profile measurements.

To study the microstructure of the hardened cement paste samples after sulphate ingress, a tungsten filament scanning electron microscope (SEM, Hitachi FlexSEM 1000, BSE detection, 15.0 kV, working distance 10 mm) was used. BSE images and elemental maps from energy-dispersive X-ray spectroscopy (EDS, Oxford Instruments AZtecOne 30 mm^2^) were obtained on polished thin sections (*d* = 20 μm, water-free preparation on glass sample holders, no sputter coating) of hollow cylinder cross-sections that were dried 30 ${}^{{}^{\circ}}C$ and ambient pressure for 72 h after the pore solution was replaced by isopropyl alcohol (the positioning of the samples is illustrated in Figure 3). To avoid charging, the SEM was operated at a chamber pressure of 30 Pa.

The distribution of sulphur within the cross-section of selected hollow cylinders was measured by laser ablation inductively coupled plasma mass spectrometry (LA-ICP-MS, the positioning of the samples is illustrated in Figure 3). Segments of hollow cylinder cross-sections were cut using a precision saw (Buehler IsoMet 5000). Afterwards, the cross-section segments were dried at 30 ${}^{{}^{\circ}}C$ and ambient pressure for 72 h in a desiccator with silica gel. This drying procedure was chosen to minimise the decomposition of ettringite [20]. The LA-ICP-MS measurements followed a procedure that is well proven for hardened cement paste samples [21]. An ESI NWR 213 Nd:YAG laser with a two-volume cell system operating at a wavelength of 213 nm was used for laser ablation. To expel air and moisture, the sample chamber was purged with helium for 5 min. Dust and residues from sample preparation on the sample surfaces were removed by initially ablating with a 80 μm radiation spot at a scan velocity of 60 μm s^−1^. To obtain the actual sulphur content profiles, ablation was conducted along defined straight lines consisting of a series of 40 μm spots. The lines were shot with a frequency of 20 Hz, a flux density of 2.1 J cm^−2^ and a scan velocity of 10 μm s ^−1^. The ablated material was transported by a helium flux (0.7 L min${}^{-1}$) to an ICP-MS apparatus (Perkin Elmer NexION 300D, torch power 1200 W). The isotope ^34^S was recorded to obtain the sulphur profiles. Since an established calibration procedure for LA-ICP-MS on hardened cement paste is not currently available, the results are presented as raw data (counts per second) and should therefore be regarded as semi-quantitative.

### 2.3. Finite Element Modelling

A finite element modelling approach was chosen to study the effect of crystallisation pressure caused by the formation of ettringite and/or gypsum on the stress distribution within the undamaged inner core of the sample. The approach is based on the crystallisation pressure theory [3,22,23]. When external sulphate enters the pore system of mortar or hardened cement paste, the formation of ettringite and/or gypsum will occur in the outer parts of the samples first [15] and will induce expansive stress in the undamaged core [5], which can exceed the tensile strength of the binder matrix [4]. Assuming that the pore solution in pores with a certain diameter at a certain depth reaches the same supersaturation with respect to ettringite and/or gypsum (the sulphate ingress front is parallel to the samples surface), the expansive crystallisation of ettringite and/or gypsum will occur at this specific sample depth after a given storage duration. This leads to the formation of an expansion front that moves towards the sample’s core over time and exerts pressure on the samples inner, undamaged core.

The modelling was conducted using the Ansys software package (v.2021 R2, Ansys, Inc., Canonsburg, PA, USA). The expansion front was defined as the curved surface of the undamaged inner core of the hollow cylinder. Expansion fronts moving from the inner and outer surfaces of the hollow cylinders were simulated. In order to consider the effect of larger expansion front depths, the wall thickness of the undamaged inner core was decreased accordingly. The end surfaces of the hollow cylinders were defined as fixed support, which corresponds approximately to the longitudinal constraint that was exerted by the central steel rods. The stress pattern within the undamaged inner core of the hollow cylinders was visualised for the largest primary stress. The pressure that is exerted within the expansion front depth on the sample’s inner core was set to 8 MPa, as this value has been observed for both larger and lower sulphate concentrations in hardened cement paste made with Portland cement ([4,14], and the results presented here). The mechanical properties of the hardened cement paste were based on experimental data for samples prepared with OPC M after a storage duration of 100 days in saturated calcium hydroxide solution since the undamaged inner core of the sample was not altered by the sulphate ingress (compressive strength: 60.7 N mm^−2^; dyn. Young’s modulus: 23.7 GPa; bulk density: 1840 kg m^−3^). The Poisson ratio was estimated at 0.26 [24].

### 2.4. Estimating the Maximum Crystallisation Pressure

In order to estimate the maximum crystallisation pressure that can be thermodynamically supported in the hardened cement paste, thermodynamic modelling of the saturation indices (SI) of ettringite and gypsum was performed. The GEM-Selektor (GEMS) software package (v.3.7.0, [25,26]) was used for modelling, and the thermodynamic parameters were taken from the Cemdata database (v.18.1, [27]). The input phase data were taken from Table 1, and the mass of water calculated according to the water/binder ratio of 0.5. The temperature for all thermodynamic calculations was set to 22 ${}^{{}^{\circ}}C$ according to the experimental conditions in the laboratory, and therefore, the crystallisation of thaumasite was suppressed in the modelling because its formation is usually observed at lower temperatures only [28]. The crystallisation of quartz, hematite, goethite, kaolinite, graphite, magnetite, pyrite, troilite and pyrolusite was also excluded because their formation is kinetically unlikely for the chosen experimental conditions. Changes in the phase assemblage due to sulphate ingress were modelled by the addition of an increasing volume of the sodium solution with a constant sulphate concentration (3.0 g L $SO_{4}^{2-}\;\mathrm{or}\;30.0g\;L$ SO_4_^2−^). The maximum saturation indices of ettringite or gypsum were estimated in separate modellings with further crystallisation restrictions (SI of ettringite: ettringite content limited to the amount of primary ettringite, crystallisation of monosulfoaluminate suppressed; SI of gypsum: formation of gypsum and monosulfoaluminate suppressed). The maximum saturation indices log(Q/K) of ettringite and gypsum were then used to calculate the maximum crystallisation pressure *p* (Pa) according to the Correns Equation (Equation (2), [3,11]), in which $R_{g}$ is the universal gas constant (8.314 J K^−1^ mol^−1^), *T* is the temperature (295 K) and $V_{C}$ ($cm^{3}$ mol^−1^) ist the molar volume of the crystalline phase. $V_{C}$ of ettringite was set to 707 $cm^{3}$ mol^−1^ [4] and $V_{C}$ of gypsum was set to 298 $cm^{3}$ mol^−1^ (calculated from the cell volume of 494.4 ${Å}^{3}$ [29]).

(2) $$p=\frac{R_{g}T}{V_{C}}\;\cdot \;\ln\left(\frac{Q}{K}\right)$$

## 3. Results and Discussion

### 3.1. Comparison of Maximum Crystallisation Pressure and Maximum Expansion Stress

The results of the thermodynamic estimation of the maximum crystallisation pressure (Equation (2)) for the formation of ettringite and gypsum are shown in Table 2. The results for ettringite are in good agreement with literature data for similar phase assemblages [6]. The maximum crystallisation pressure for ettringite is not significantly affected by the sulphate concentration of the sodium sulphate solution, indicating that the maximum pressure is primarily controlled by the availability of $Ca^{2+}$ in the pore solution. The maximum crystallisation pressure for ettringite is even higher at 3.0 g L SO_4_^2−^ compared to 30.0 g L $SO_{4}^{2-}$, most probably because smaller amounts of Ca^2+^ are bound in gypsum at the lower sulphate concentration. This agrees with the results of thermodynamic calculations of the phase assemblage and $Ca^{2+}$ concentration of the pore solution for hardened Portland cement paste that is in equilibrium with sodium sulphate solutions of various concentrations reported in [10].

However, these maximum crystallisation pressures, derived from thermodynamic modelling results, are unlikely to occur in real samples because the transport and mobility of the reactants that form ettringite is restricted. Furthermore, pores only represent a fraction of the sample’s cross-sectional area, and therefore, the crystallisation pressure is not equally distributed over the whole binder matrix. This means that the maximum crystallisation pressure cannot act within the whole cross-sectional area (*A* in Equation (1)) since the pores cover only a fraction of the overall cross-sectional area of the hollow cylinder. Therefore, the expansion stress in hardened cement paste would most likely never reach the thermodynamically predicted maximum crystallisation pressure even after a long time.

Due to the limited transport rate of sulphate, real crystallisation pressures caused by the crystallisation of ettringite are likely to be larger in samples exposed to solutions with 30.0 g L $SO_{4}^{2-}$ than lower sulphate concentrations because the ingress of a storage solution with higher sulphate concentrations can lead to increased sulphur content in the binder matrix more rapidly (e.g., [5]). Despite the high sulphate concentration of the exposure solution applied in this study, the thermodynamically estimated maximum crystallisation pressures for ettringite are, however, still much larger than the maximum expansion stress observed in the hollow cylinder method after storage in 30.0 g L SO_4_^2−^, as mentioned above and in [4,14]. Although it is apparent that the expansion stress in hardened cement paste might never reach the thermodynamically predicted maximum crystallisation pressure, it remains unclear whether the actual maximum expansion stress in hollow cylinders is limited by the sample geometry, relaxation due to creep and the (lack of) stiffness of the hardened cement paste itself.

The maximum crystallisation pressure of gypsum is much smaller than for ettringite and is zero for hardened cement paste made with OPC H after storage in 3.0 g L $SO_{4}^{2-}$ since no stable gypsum is thermodynamically predicted at this sulphate concentration for this binder. These results correlate with experimental studies indicating that gypsum might—if at all—contribute to the degradation of the binder matrix at very large sulphate concentrations only [7,9].

### 3.2. Testing the Condition σ=(k/A)Δl

**Figure 4:**
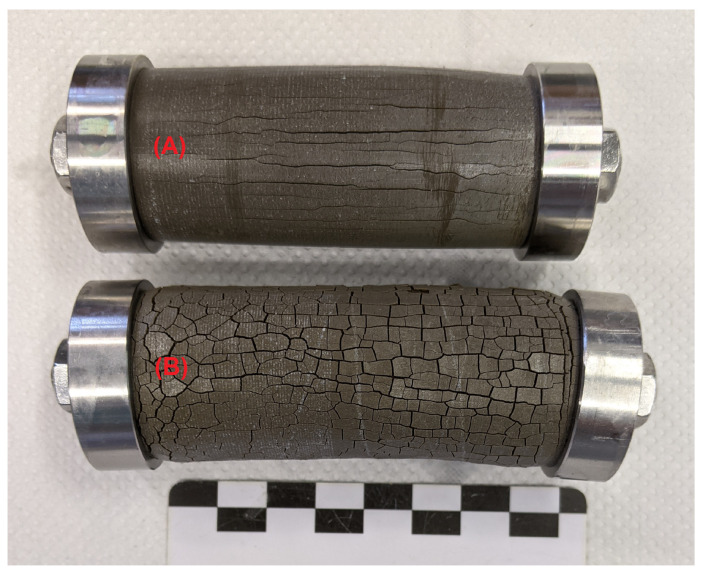
Characteristic crack patterns of hollow cylinder samples after storage in sodium sulphate solution: (**A**) stress cell with additional constraint by a central steel rod: major cracks are formed among the longitudinal axis; (**B**) stress cell with virtually no additional constraint by using a steel spring with a low spring constant, where a network pattern of cracks is formed due to equal elongation of the sample in every spatial direction.

**Figure 5:**
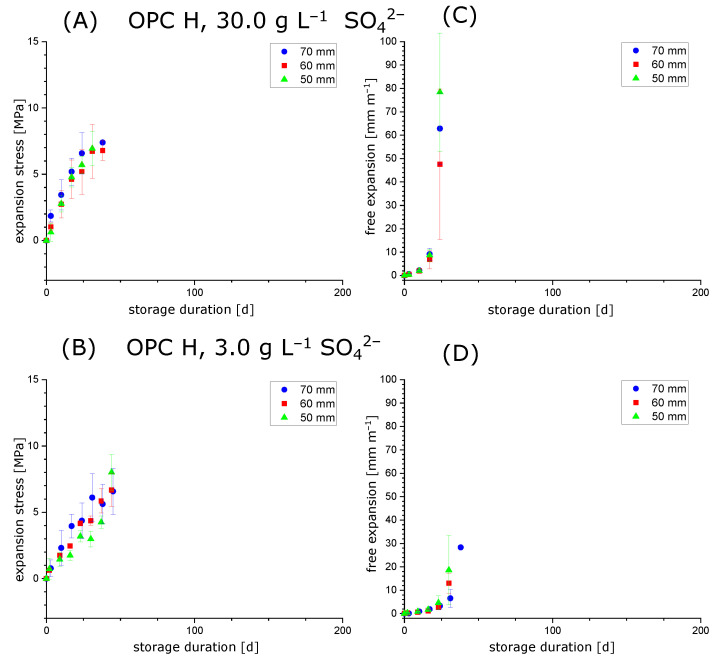
Expansion experiments (sodium sulphate solution, 30.0 g L $SO_{4}^{2-}\;\mathrm{or}\;3.0g\;L$ SO_4_^2−^) for hollow cylinders with varying diameter/length ratio prepared with OPC H. The samples were stored until their mechanical failure due to the formation of macroscopic cracks. (**A**,**B**) Expansion stress (stress cell with steel rod). (**C**,**D**) Free expansion (stress cell with steel spring).

**Figure 6:**
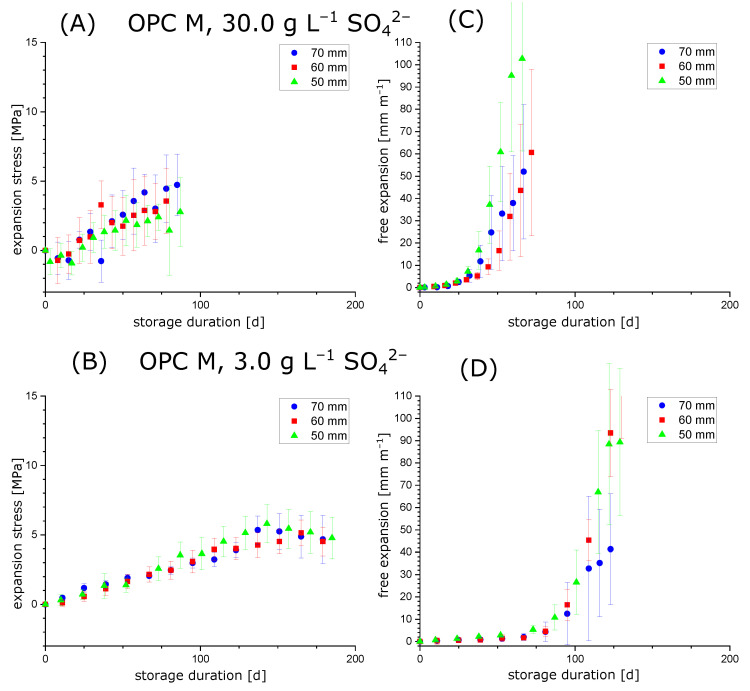
Expansion experiments (sodium sulphate solution, 30.0 g L $SO_{4}^{2-}\;\mathrm{or}\;3.0g\;L$ SO_4_^2−^) for hollow cylinders with varying diameter/length ratios prepared with OPC M. The samples were stored until their mechanical failure due to the formation of macroscopic cracks. (**A**,**B**) Expansion stress (stress cell with steel rod). (**C**,**D**) Free expansion (stress cell with steel spring).

When conducting experiments with hollow cylinders to measure the expansion stress in the samples (constrained stress cells, Figure 1), the hollow cylinders will eventually fail due to macroscopic cracking. In general, it was observed that hollow cylinders in constrained stress cells showed longitudinal cracks that comprised the whole cylinder wall (Figure 4A; in contrast, samples without additional constraint showed a network pattern of cracks, Figure 4B). This behaviour indicates that the maximum expansion stress might be limited by the samples stiffness because the hollow cylinder would be plastically deformed perpendicular to the longitudinal axis. Therefore, experiments with varying hollow cylinder length (50 mm, 60 mm and 70 mm) were performed to test whether the condition stated in Equation (1) is affected by a varying diameter/length ratio of the hollow cylinders. The cross-sectional area *A* was kept equal for all hollow cylinders, so the diameter/length ratio should not affect the expansion stress if the stiffness of the cylinder walls is large enough to withstand forces perpendicular to the longitudinal axis. The results for the hollow cylinder method with hardened cement paste prepared with OPC H or OPC M are shown in Figure 5 and Figure 6, respectively.

The experiments showed that the development of expansion stress and free expansion does not—for the range covered in this study—depend systematically on the diameter/length ratio of the hollow cylinders. This indicates that the condition in Equation (1) is valid for a certain range of hollow cylinder lengths with a constant cross-sectional area *A*. This observation suggests that the relaxation behaviour of the hardened cement paste perpendicularly to the direction of constraint is similar for the hollow cylinder length range covered in this study. This supports the assumption that the expansion stresses measured by the hollow cylinder method could be representative of hardened cement paste exposed to sulphate solutions regardless of its specific sample geometry.

As expected, samples prepared with OPC H (12 wt.% C_3_A) developed arge free expansions and expansion stress faster compared to samples prepared with OPC M (6.5 wt.% C_3_A). However, it was not possible to observe higher maximum expansion stresses after storage in 30.0 g L $SO_{4}^{2-}$ (Figure 5A and Figure 6A) compared to samples that were stored in 3.0 g L SO_4_^2−^ (Figure 5B and Figure 6B) for both cements. On the one hand, this correlates qualitatively with the thermodynamic estimations of the maximum crystallisation pressures (Table 2), but on the other hand, this phenomenon could possibly be caused by the fast degradation rate of the hollow cylinders stored in 30.0 g L $SO_{4}^{2-}$: if the degradation of samples is too fast, the actual maximum expansion stress and maximum free expansion might be missed between two measurements, which were performed every 7 days. However, the maximum expansion stresses observed experimentally are still about an order of magnitude smaller than the estimated maximum crystallisation pressures for both sulphate concentrations, as explained in Section 2.4.

### 3.3. Effect of the Hollow Cylinders Curvature on the Stress Distribution

**Figure 7:**
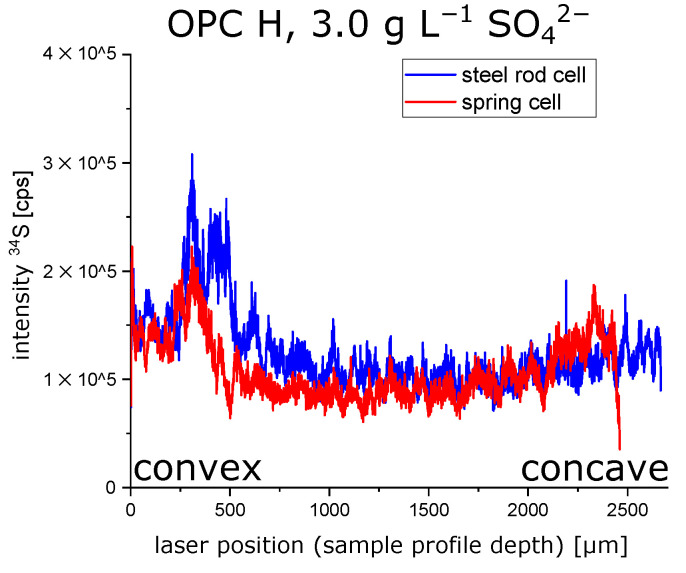
LA-ICP-MS profiles of the sulphur content (solid phases) over the cross-section of hollow cylinder walls (OPC H) at the time of mechanical failure of the samples after 38 days (non-constrained stress cell) and 59 days (constrained stress cell) of storage in 3.0 g L $SO_{4}^{2-}$, respectively.

**Figure 8:**
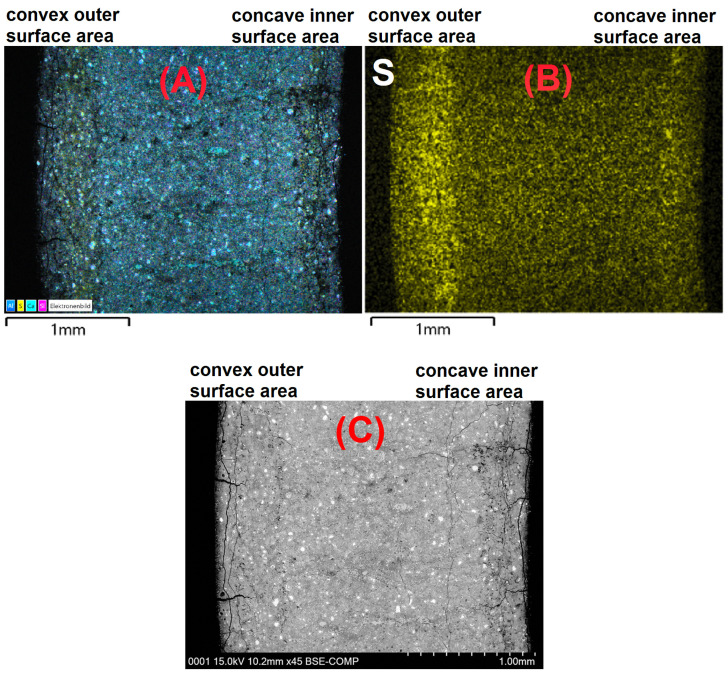
Overlay map (**A**) of a BSE image (**C**) with the EDS element maps of calcium, silicon, aluminium and sulphur and the EDS elemental map of the sulphur content ((**B**), solid phases) over the cross-section of hollow cylinder walls (OPC H) at the time of mechanical failure of the samples after 59 days (constrained stress cell) of storage in 3.0 g L $SO_{4}^{2-}$.

**Figure 9:**
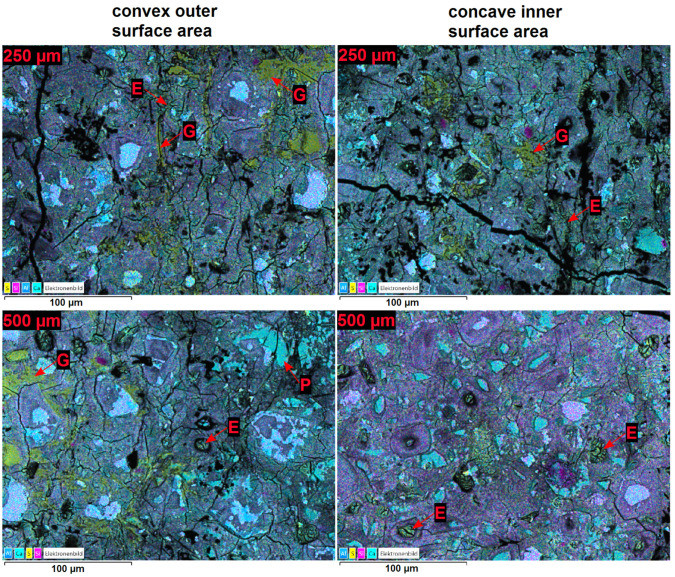
Overlay maps of BSE images with the EDS element maps of calcium, silicon, aluminium and sulphur of the microstructure over the cross-section of hollow cylinder walls (OPC H) at the time of mechanical failure of the samples after 59 days (constrained stress cell) of storage in 3.0 g L $SO_{4}^{2-}$. The sample depth was 250 μm or 500 μm, respectively. The following phases are indicated in the figure: E: ettringite; G: gypsum; P: portlandite.

LA-ICP-MS measurements were performed to obtain profiles of the sulphur content over the cross-section of a hollow cylinder wall (prepared with OPC H and stored in 3.0 g L $SO_{4}^{2-}$). The results are shown in Figure 7. Due to the removal of the pore solution by isopropyl alcohol, the sulphur content shown in LA-ICP-MS profiles is assumed to represent the sulphur content of the solid phases. The sulphur profiles show that the sulphur content maximum beneath the outer convex surface is far more pronounced than the sulphur maximum beneath the concave inner surface (Figure 7). This observation was confirmed by SEM/EDS measurements of the sulphur content over the cross-section of hollow cylinder walls (Figure 8). Despite the exposure of both the inner and outer surfaces of the hollow cylinders to the sulphate solution, larger amounts of gypsum were observed beneath the convex outer surface compared to the concave inner surface (Figure 9). One geometric property that could possibly contribute to this asymmetric sulphur content in the solid phase might be the effect of an increasing radial flux density of ions towards the samples core [30] because the flux vectors converge underneath the convex outer surface.

**Figure 10:**
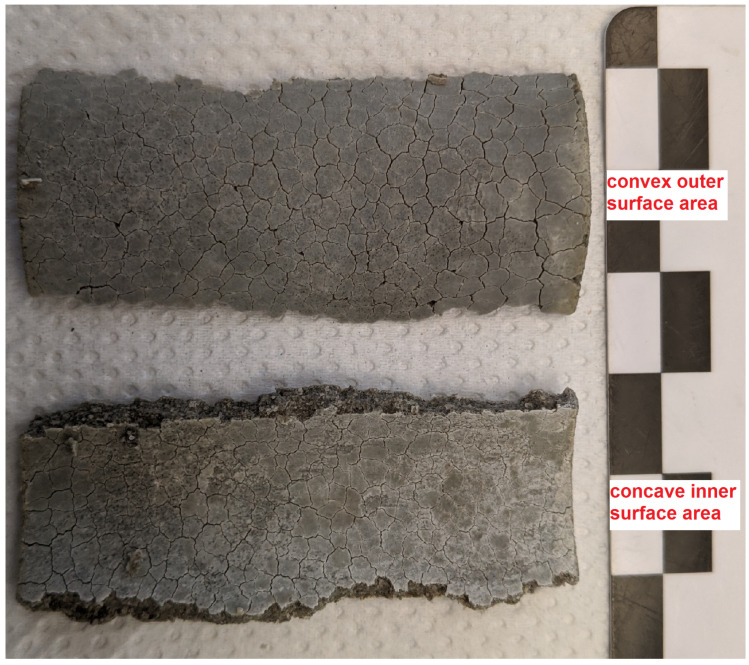
Segments of a hollow cylinder (OPC H) at the time of mechanical failure after 38 days (non-constrained stress cell) of storage in 3.0 g L $SO_{4}^{2-}$.

**Figure 11:**
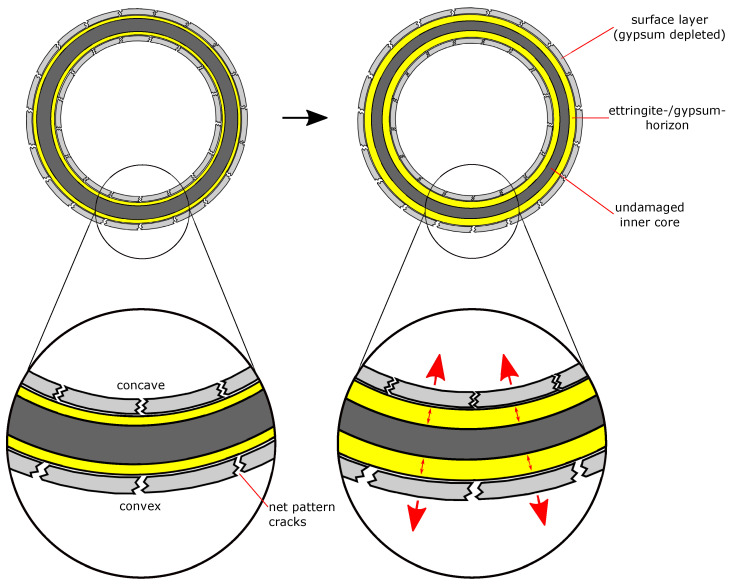
Schematic representation (not to scale) of the cross-section of a hollow cylinder that experiences an ongoing expansive formation of ettringite and/or gypsum beneath the surfaces due to the ingress of sulphate ions. The concave inner surface has a higher degree of restraint compared to the convex outer surface.

The SEM/EDS observations showed that the increased sulphur content beneath the convex outer surface is mainly caused by the presence of larger amounts of gypsum compared to the concave inner surface (Figure 9). This suggests that the gypsum content of the samples is more asymmetric than the ettringite content. This observation indicates another possible explanation of the asymmetric sulphur distribution within the hollow cylinder’s wall. It has been observed in the past that the formation of gypsum during external sulphate attack can be reduced under confined conditions more than the formation of ettringite [4]. Thus, the asymmetric gypsum distribution could mean that the degree of constraint is somewhat smaller beneath the convex outer surface compared to the concave inner surface. The form of the crack patterns in the different surfaces supports this assumption. At the time of mechanical failure, the convex outer surfaces exhibit a crack pattern with wider cracks compared to the crack pattern of the concave inner surfaces (Figure 10). These different crack patterns are a result of the formation of expansive phases beneath the surfaces. For the convex outer surface, the underlying formation of ettringite and/or gypsum can widen the surface cracks, but the effect would be inverted for the concave inner surface (Figure 11). This has two implications: First, the widening of cracks in the convex outer surface leads to an increased transport of sulphate solution towards the inner core region. Secondly, the geometric self-constraint beneath the concave inner surface increases the mechanical restraint at this position.

**Figure 12:**
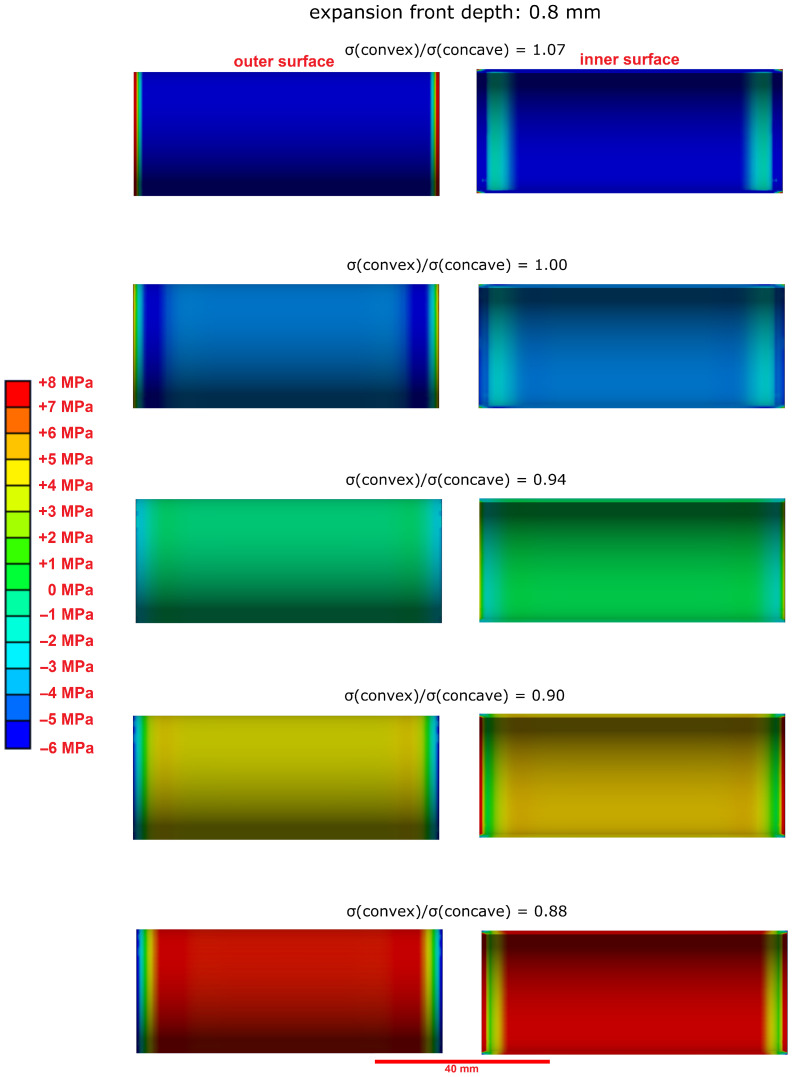
Modelled stress distribution (finite element model, first main tension) in the undamaged inner core over the length of a hollow cylinder that is exposed to expansion stress (σ(concave) = 8.0 MPa). (**Left**): at the outer surface, (**Right**): at the inner surface of the cylinder. The expansion front depth of the ettringite formation was set to 0.8 mm. A slightly larger expansion stress beneath the concave inner surface leads to tensile stress within the sample’s core that can exceed its tensile strength.

**Figure 13:**
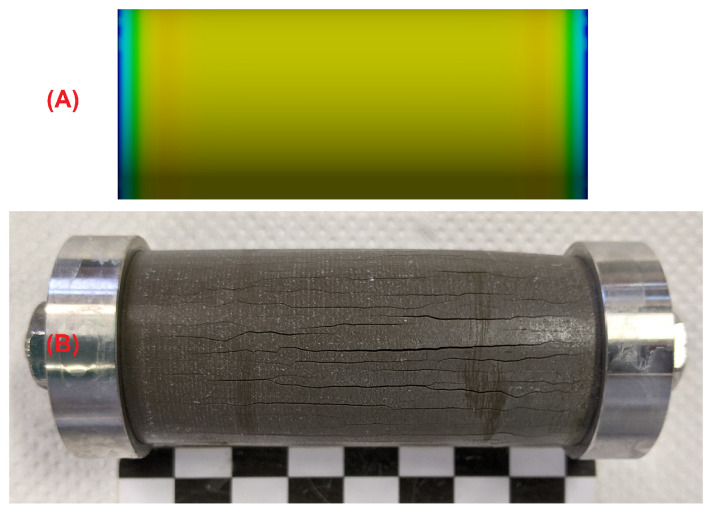
Modelled stress distribution ((**A**), finite element model, first main tension) in the undamaged inner core of a hollow cylinder (*l* = 70 mm) that is exposed to expansion stress (σ(concave) = 8.0 MPa, σ(convex) = 7.2 MPa). The tensile strength of the sample’s inner core is exceeded, thus causing the formation of longitudinal macroscopic cracks, as observed in the experiments (**B**).

The observation of longitudinal cracks in hollow cylinders that were constrained by a central steel rod (Figure 4A) shows that the tensile strength of the undamaged inner core of the hollow cylinder has been surpassed at some point of the sulphate ingress. This cannot be readily explained by the effect of equal expansion stresses beneath the outer and inner surfaces of the hollow cylinder because the the pressure from both surfaces would lead to an even compressive load on the sample’s inner core. The compressive strength of hardened cement paste is much larger than its tensile strength, and therefore, this evenly distributed compressive load would not necessarily lead to the observed mechanical failure of the hollow cylinders. However, the failure behaviour of hollow cylinders indicates that the concave inner surface region of hollow cylinders are able to support larger expansion stresses due to a larger self-constraint compared to the convex outer surface region. A finite element modelling approach was chosen to visualise the effect of such an asymmetric self-constraint. The LA-ICP-MS profiles of the sulphur content of hollow cylinder wall sections showed the formation of ettringite following the sulphate ingress up to a sample depth of about 0.8 mm (Figure 7). The results of the finite element modelling showed that a slightly larger expansion stress beneath the concave inner surface (8.0 MPa vs. 7.2 MPa beneath the convex outer surface) led to the formation of significant tensile stress within the undamaged sample core (Figure 12). This tensile stress exceeded the tensile strength of the undamaged hardened cement paste (about 3.0 MPa) and therefore leads to the formation of longitudinal macroscopic cracks, as observed in the experiments (Figure 13).

**Figure 14:**
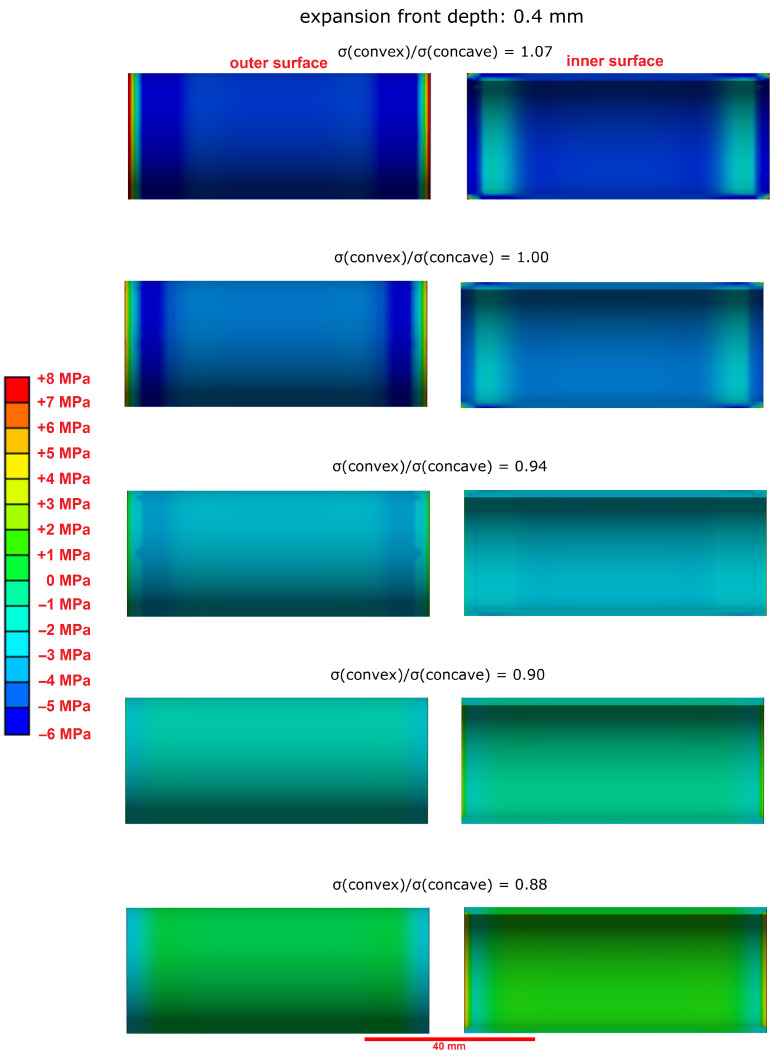
Modelled stress distribution (finite element model, first main tension) in the undamaged inner core over the length of a hollow cylinder that is exposed to expansion stress (σ(concave) = 8.0 MPa). (**Left**): at the outer surface, (**Right**): at the inner surface of the cylinder. The expansion front depth of the ettringite formation is 0.4 mm. The tensile strength of the samples inner core is not exceeded for this expansion front depth.

The modelling results also suggested that the asymmetric expansion stress must reach a certain sample depth because no sufficient tensile stress was induced in the sample’s inner core for an expansion front depth of 0.4 mm (Figure 14). This correlates with observations of other studies, which suggested that the volume ratio of the damaged outer samples area to the undamaged core must reach a certain value before the tensile strength of the undamaged core is exceeded [5,15].

These results indicate that the maximum overall expansion stress produced by the hollow cylinder method is limited by the tensile strength of the undamaged inner core of the hollow cylinder. Owing to the cylindrical geometry, the tensile strength is exceeded by stresses perpendicular to the longitudinal cylinder axis. This probably explains the similarity of the maximum observed expansion stresses for different binders and sulphate concentrations. Stress relaxation by creeping is also likely to contribute to the underestimation of expansion stress measured with the hollow cylinder method. It should be pointed out that larger expansion stresses could occur in real building structures that have higher self-constraint due to their weight and a higher tensile strength of reinforced concrete. The measurement of expansion stress with newly designed stress cells that use thicker cylinder walls and some degree of constraint perpendicular to the longitudinal axis could therefore elucidate the upper limit of expansion stress during an external sulphate attack.

## 4. Conclusions

In the present study, hollow cylinder expansion experiments, microstructural analysis and finite element modelling were carried out to reveal new insight into the degradation mechanisms of hardened Portland cement paste cylinders during an external sulphate attack on concrete structural components. The validity of the expansion stress calculated from constrained expansion was verified using hollow cylinders with different diameter/length ratios. The following conclusions can be made based on the presented results:

- Thermodynamically estimated crystallisation pressures for the formation of ettringite are much larger than the expansion stress observed experimentally in constrained hollow cylinders using stress cells. The stress produced by the crystallisation of ettringite or gypsum is affected by sample geometry, degree of restraint, pore sizes in which crystallisation occurs and relaxation processes.
- The microstructural analysis and the macroscopic crack patterns of failed hollow cylinders indicate a higher degree of self-constraint beneath the concave inner surface compared to the convex outer surface when ettringite and/or gypsum are formed following the ingress of sulphate. Finite element modelling results indicate that this asymmetric self-constraint leads to tensile stresses in the inner core of the hollow cylinder and thus longitudinal cracks and failure. Hardened cement paste with lower tensile strength can therefore possibly support smaller maximum expansion stresses compared to samples with larger tensile strength when using the hollow cylinder method.
- Real building structures are likely to accommodate larger expansion stresses during an external sulphate attack than observed with the hollow cylinder method due to additional self-constraint in heavy structural components and the higher tensile strength of reinforced concrete. Like in the experiments used in this study, the maximum expansion stress in real building structures is most likely smaller than the thermodynamically predicted maximum crystallisation pressure, but this requires further verification. New long-term studies to quantify the expansion stress in concrete under field conditions would be required to elucidate this parameter. These studies would require a new experimental setup since the hollow cylinder method cannot be adapted for concrete due to the small wall thickness and the effect of asymmetrical self-constraint below the cylinder surfaces described in this study.

## Figures and Tables

**Table 1:** Chemical composition (ICP-OES) and mineralogical composition (XRD) of the binders used in this study (wt.%).

Element	OPC H	OPC M		Phase	OPC H	OPC M
LOI	1.90	2.96		C_3_S	64	64
$Na_{2}O$	0.25	0.49		C_2_S	12	12
$K_{2}O$	0.84	1.14		C_3_A	12	6.5
CaO	63.70	63.16		C_4_AF	4	8.5
MgO	1.38	2.12		Gypsum	n.d.	n.d.
$Fe_{2}O_{3}$	2.27	2.67		Bassanite	3	n.d.
$Al_{2}O_{3}$	5.25	4.50		Lime	n.d.	1
$SiO_{2}$	20.80	19.72		Periclase	n.d.	2
$P_{2}O_{5}$	0.26	0.29		Quartz	<1	n.d.
$SO_{3}$	2.78	3.46		Calcite	3	3
$TiO_{2}$	0.28	0.26		Arcanite	n.d.	1
MnO	0.06	0.03		Anhydrite	n.d.	2
∑ elements	99.77	100.80		∑ phases	99	100

**Table 2:** Thermodynamic estimation of the maximum crystallisation pressure [MPa] for the formation of ettringite and gypsum in hardened cement paste exposed to sodium sulphate solution.

			Ettringite				Gypsum	
Sulfate Concentration		OPC H		OPC M		OPC H		OPC M
30.0 g L $SO_{4}^{2-}$		53.4		53.4		5.3		4.7
3.0 g L $SO_{4}^{2-}$		54.1		54.1		0.0		1.2

## Data Availability

The data presented in this study are available on request from the corresponding author.
